# Eclectic Department

**Published:** 1856-03

**Authors:** 


To the Editor of the N. Y. Daily Times.

*A Rare Blessing.*—I call upon you as a friend of humanity, to give place to the following information in the columns of your invaluable paper, *pro bono publico:*

I am the inventor of a *new kind* of medicine which will cure *every disease* by operating on the *nervous fluid* and the *cords*. This, it will be perceived, differs from Dr. Brandreth's Pills, and reaches far beyond them, as his medicine operates merely on the blood, leaving surgical matters entirely out of the question; while my medicine works in a double sense, medical and surgical. I do not deem it expedient, at present, to state *how* my *Nervine* acts in setting dislocated bones, &c., by its constricturing determinability upon the cords, muscles, &c., of the body; but shall leave any further statements until I shall have filed a caveat; and in the meantime it sufficeth to say: That I have discovered in one of our common plants, growing indigenously in our corn-fields, the most stupendous and transcendant medicinal qualities, and which will cure all kinds of disease, almost spontaneously.

I have made a medicine from this plant and conducted some experiments with it, and the following is the result:

One bottle will cure a common pathology!

One to one and a half will cure diagnosis prognosticus!

One to three will cure *habeas corpus!*

One to four will cure swelled head!

Four bottles will cure thunder humor!

Six to eight bottles will cure consumption, even if the lungs are gone!

And nine bottles will cure aurora borealis!!

To cure a broken bone, wash the parts well with the medicine.

A sprain is cured by rubbing the empty bottle over the part affected.

U. B. GASID, M. D.

P. S.—My laboratory and wholesale warerooms are in Hartford, Conn.; also my residence. Persons wishing my medicine will address accordingly. Price, $1 per bottle; to the clergy half price.

